# Focus on Nintedanib in NSCLC and Other Tumors

**DOI:** 10.3389/fmed.2016.00068

**Published:** 2016-12-19

**Authors:** Anna Manzo, Guido Carillio, Agnese Montanino, Raffaele Costanzo, Claudia Sandomenico, Gaetano Rocco, Alessandro Morabito

**Affiliations:** ^1^Thoracic Medical Oncology, Istituto Nazionale Tumori, “Fondazione G. Pascale” – IRCCS, Napoli, Italy; ^2^Department of Oncology and Hematology, Azienda Ospedaliera Pugliese-Ciaccio, Catanzaro, Italy; ^3^Thoracic Surgery, Istituto Nazionale Tumori, “Fondazione G. Pascale” – IRCCS, Napoli, Italy

**Keywords:** nintedanib, angiogenesis inhibitors, VEGF, NSCLC, review

## Abstract

Nintedanib is a new triple angiokinase inhibitor that potently blocks the proangiogenic pathways mediated by vascular endothelial growth factor receptors, platelet-derived growth factor receptors, and fibroblast growth factor receptors. Evidence about its efficacy in addition to second-line chemotherapy in non-small cell lung cancer (NSCLC) has been produced by two large randomized phase III clinical trials (LUME-Lung 1 and LUME-Lung 2), conducted in patients with pretreated NSCLC, without major risk factors for bleeding. In the LUME-Lung 1, the addition of nintedanib to docetaxel significantly improved progression-free survival, which was the primary end point of the trial (3.4 vs. 2.7 months, hazard ratio: 0.79; *p* = 0.0019). Furthermore, a significant improvement in median overall survival (from 10.3 to 12.6 months) was observed in patients with adenocarcinoma histology, with a greater advantage in patients who progressed within 9 months after start of first-line treatment (from 7.9 to 10.9 months) and in patients who were most refractory to first-line chemotherapy (from 6.3 to 9.8 months). Adverse events were more common in the docetaxel plus nintedanib group, and they included diarrhea and increased liver enzymes, while no statistically significant increase in the incidence of bleeding and hypertension events by the addition of nintedanib was observed. On these bases, the combination of docetaxel and nintedanib can be considered a new option for the second-line treatment for patients with advanced NSCLC with adenocarcinoma histology. Future challenges are the identification of predictive factors to help the decision of using nintedanib in eligible patients.

## Introduction

In recent years, a better understanding of the biology of cancer led to the development of molecular targeted therapies that have radically changed the treatment of many solid tumors, including non-small cell lung cancer (NSCLC). The new tailored agents, such as epidermal growth factor receptor (EGFR) tyrosine kinase inhibitors (TKIs) and anaplastic lymphoma kinase inhibitors, are able to inactivate specific molecular alterations that occur in specific oncogenes, which cause cancer cell survival strictly dependent on such aberrant genes, as explained by the “oncogene addiction theory” (1). However, only a minority of tumors are oncogene addicted, and chemotherapy remains the only treatment available for the majority of cancer patients.

In this setting, targeting the angiogenesis pathways represents an alternative and attractive strategy, inasmuch as tumor development, progression, and metastasis are demonstrated strongly linked to angiogenesis. Angiogenesis is a very complex process, which is highly regulated by many molecules with both proangiogenic and antiangiogenic activity. The tumor microenvironment is composed of hyperproliferating cells that need large amounts of oxygen and nutrients. Such cells are able to deregulate the angiogenic process inducing an abnormal secretion of proangiogenic factors and the consequent development of disorganized, tortuous, enlarged, high permeable blood vessels, which are needed for both tumor growth and its metastatic potential (2). Therefore, angiogenic pathways have been investigated as potential therapeutic targets in patients with NSCLC (3). Several antiangiogenic agents have been developed, including monoclonal antibody anti-vascular endothelial growth factor (VEGF) such as bevacizumab or vascular endothelial growth factor receptor (VEGFR) TKIs, such as sorafenib and sunitinib. In particular, bevacizumab in combination with platinum-based chemotherapy has demonstrated superior efficacy compared with chemotherapy alone as first-line treatment in patients with non-squamous NSCLC, reaching the approval for use in this setting (4). However, because of substantial redundancy of proangiogenic pathways, patients treated with bevacizumab inevitably develop resistance to this agent (3).

One strategy for overcoming acquired resistance to bevacizumab is to target simultaneously multiple angiogenic receptors. Nintedanib is a new triple angiokinase inhibitor that potently blocks the proangiogenic pathways mediated by VEGF receptors, platelet-derived growth factor (PDGF) receptors, and fibroblast growth factor (FGF) receptors. This review summarizes the clinical data emerging from phase I–III clinical studies with nintedanib in NSCLC and in other tumors, focusing on the data that led to the recent approval by the European Medicines Agency as a second-line treatment in association with docetaxel in patients with advanced NSCLC.

## Preclinical Evidence

**Figure 1:**
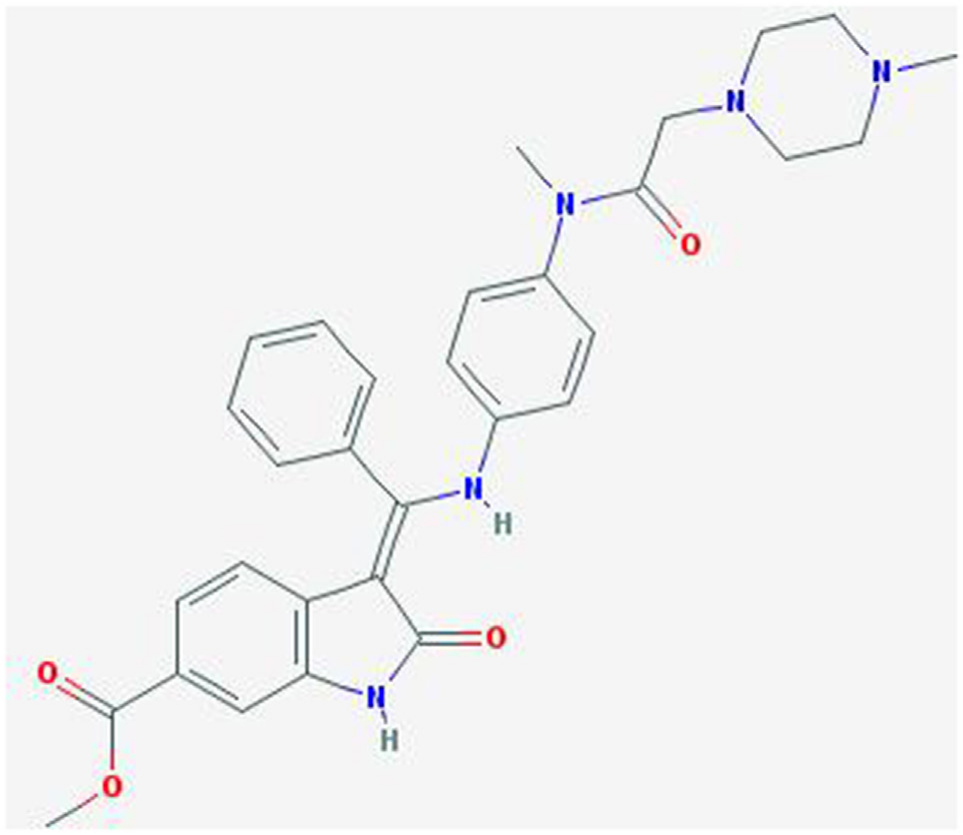
**Chemical structure of Nintedanib**.

**Figure 2:**
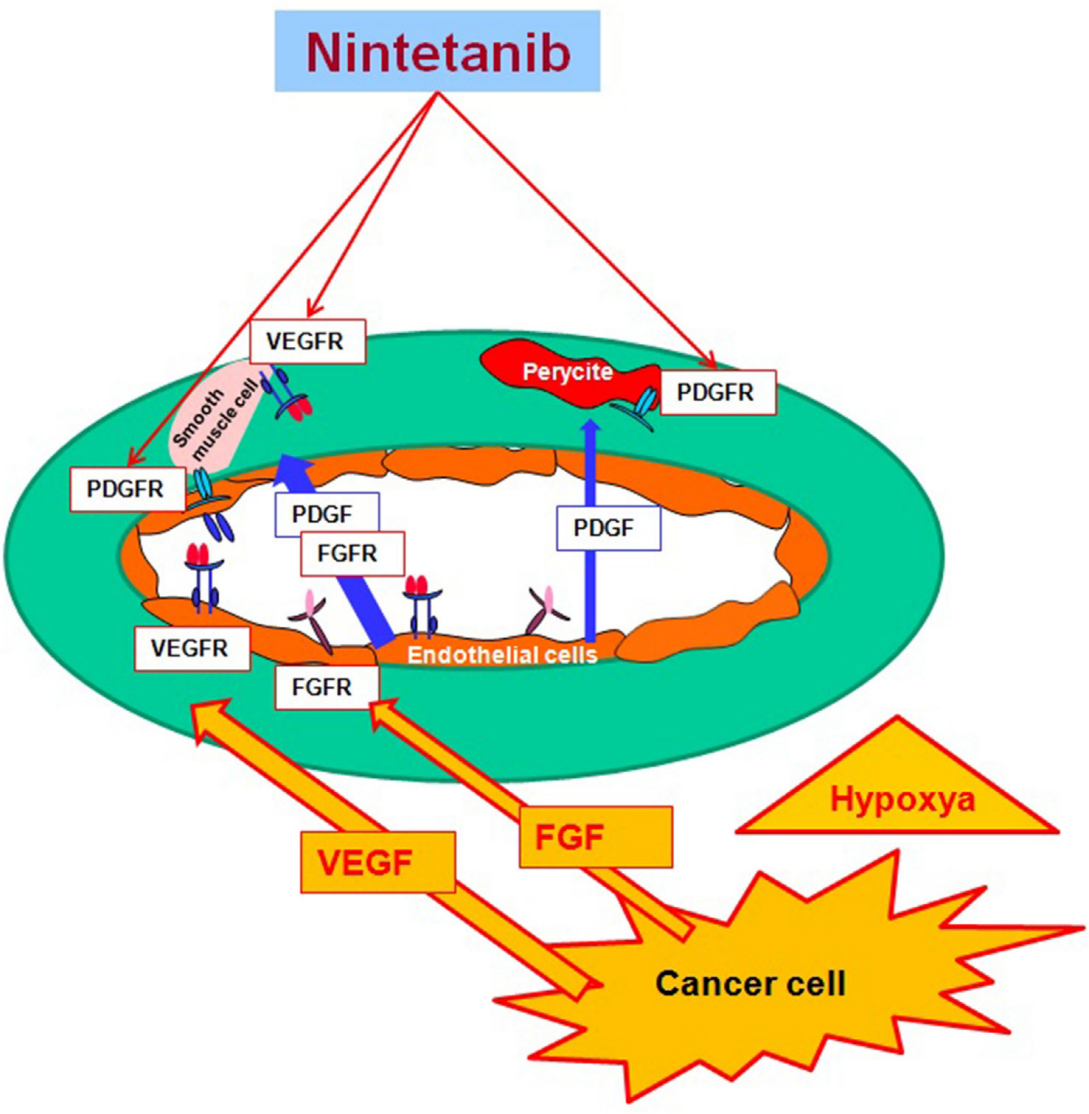
**Nintedanib and targeted proangiogenic pathway**.

Nintedanib (BIBF 1120; methyl (3Z)-3-[[4-[methyl-[2-(4-methylpiperazin-1-yl)acetyl]amino]anilino]-phenylmethylidene]-2-oxo-1H-indole-6-carboxylate) is a potent, oral angiokinase inhibitor that targets the proangiogenic pathways (Figure 1). This molecule is an indolinone derivative that blocks adenosine triphosphate-binding sites in the kinase domain of proangiogenic receptors inhibiting the downstream signaling pathways related to neoangiogenesis. Nintedanib is a TKI targeting VEGFR1–3, platelet-derived growth factor receptor α (alpha) and β (beta), and fibroblast growth factor receptors (FGFR) 1–3 and, in addition, it also inhibits the Src family, RET, and FLT3 (5, 6) (Figure 2). The three VEGF receptors have different functions, but all take part in tumorigenesis, directly stimulating cancer stem cell proliferation (6). Moreover, VEGFR-2 is considered the crucial receptor involved in initiation of the formation as well as the maintenance of tumor vasculature. Preclinical studies with nintedanib have shown sustained (>30 h) blockade of VEGFR2 *in vitro* and delay or arrest of tumor growth in xenograft models of human solid tumors, including lung cancer models (7). The specific and simultaneous abrogation of all the pathways targeted by nintedanib results in effective growth inhibition of both endothelial and perivascular cells, which may be more effective than inhibition of endothelial cell growth alone.

Furthermore, signaling by FGF receptors has been identified as a possible escape mechanism for tumor angiogenesis when the VEGF pathway is disrupted (8). Nintedanib leads to an important decrease of microvessel density and pericyte coverage, and this leads to a diminished perfusion and thereby to the death of tumor cells. In addition, a therapeutic effect may also result from inhibition of tumor autocrine and paracrine growth factor loops involving VEGF, PDGF, and bFGF.

In a preclinical study with models of lung and pancreatic cancer, it has been described that nintedanib does not increase the markers of epithelial to mesenchymal transition that usually allow tumor cells to switch from one pathway to another. This evidence is very important and could explain why this drug does not promote the change to a more aggressive tumor subtype and does not induce chemotherapy resistance (9).

Following oral administration, nintedanib is rapidly absorbed, with a median time to maximum plasma concentration of 1.3 h and a terminal half-life of 13.7 h (10). The major route of elimination of nintedanib is through metabolism, and its metabolites are excreted *via* the biliary system into the feces; urinary excretion is minor (1%). Nintedanib metabolism in healthy humans occurs predominantly by cleavage of the methyl ester moiety, yielding the carboxylate BIBF 1202 (metabolite 1). BIBF 1202 is then conjugated to glucuronic acid, yielding 1-*O*-acylglucuronide (metabolite 2). Thus, metabolism of nintedanib is predominantly cytochrome P450 enzyme independent, which facilitates the combination of nintedanib with cytotoxic chemotherapies, such as docetaxel, that are metabolized *via* cytochrome P450 enzymes (10).

Phase I, II, and III clinical trials have been conducted in NSCLC to investigate the pharmacokinetics, tolerability, and efficacy of this triple angiokinase inhibitor (Table 1).

**Table 1:** **Randomized clinical studies with nintedanib in non-small cell lung cancer (NSCLC)**.

Phase and reference	Line of treatment	Setting	#Patients	Treatment	Results

Systemic treatment					
I; Ellis et al. (11)	>1st	Advanced NSCLC	26	Nintedanib (starting dose 100 bid) days 2–21 + pemetrexed 500 mg/mq q 21	Maximum tolerated dose (MTD) 200 mg bid
					SD 50%
I; Doebele et al. (12)	1st	Advanced NSCLC	26	Nintedanib (starting 50 mg bid) days 2–21 + carboplatin AUC6 + paclitaxel 200 mg/mq q 21	MTD 200 mg bid
					PR 26.9%; SD 38.5%
II; Reck et al. (13)	≥2nd	Advanced NSCLC, any histology	73	Nintedanib 250 mg × bid or nintedanib 150 mg bid	mPFS (all patients) 6.9 weeks
					mOS: 21.9 weeks
					Overall survival (OS) 150 vs. 250 mg b.i.d., 20.6 vs. 29.7 weeks; hazard ratio (HR): 0.693; *p* = 0.21
III, LUME-Lung 1; Reck et al. (14)	2nd	Advanced NSCLC, any histology	1,314	Docetaxel 75 mg/mq q 21 + nintedanib 200 mg bid, days 2–21 vs. docetaxel 75 mg/mq q 21	RR%: 4.7 vs. 3.6
					Disease control rate%: 60.2 vs. 44, *p* < 0.0001
					Progression-free survival (PFS): 3.4 vs. 2.7 months, HR: 0.79, *p* = 0.0019
					OS:10.1 vs. 9.1 months, ^a^HR: 0.94, *p* = 0.27
III, LUME-Lung 2; Hanna et al. (15)	2nd	Advanced NSCLC non-squamous histology	713	Docetaxel 75 mg/mq q 21 + nintedanib 200 mg bid, days 2–21 vs. docetaxel 75 mg/mq	RR%: 9.1 vs. 8.3
					Disease control rate%: 60.9 vs. 53.3, *p* = 0.039
					PFS: 4.4 vs. 3.6 months, HR: 0.83, *p* = 0.04
					OS:12.2 vs. 12.7 months, HR: 1.03, *p* = 0.79

*^a^OS not statistically different for all histology, but for subgroup non-squamous histology, OS is 12.6 vs. 10.3 months*.

## Phase I Studies

Nintedanib showed a manageable safety profile and antitumor activity in patients with solid tumors, including NSCLC (13, 16). Based on several phase I dose-escalation trials of nintedanib as monotherapy, the maximum tolerated dose (MTD) of nintedanib was defined as 250 mg twice a day (b.i.d.) in Caucasian patients and 200 mg b.i.d. in Japanese patients (17, 18).

In a phase I accelerated titration study, Mross et al. investigated the MTD and tolerability of nintedanib in 61 patients with advanced cancers (16). Nintedanib showed a favorable safety profile in this advanced cancer patient population. Twice-daily dosing permitted an increase in total dose without additional toxicity. Because of its pharmacokinetic profile and absence of interaction with CYP450 enzymes, nintedanib was investigated in combination with standard cytotoxic chemotherapies, such as docetaxel or pemetrexed (11, 19, 20).

Ellis et al. investigated the MTD of continuous oral treatment with nintedanib in combination with standard-dose pemetrexed (500 mg/m^2^) (11). Doebele et al. have also investigated the safety, tolerability, and MTD of nintedanib (starting dose 50 mg b.i.d.) on days 2–21 in combination with carboplatin [area under the curve (AUC) 6 mg/ml/min] and paclitaxel (200 mg/m^2^) on day 1 of each 21-day cycle, in first-line setting in 26 patients with advanced NSCLC (12). The MTD of nintedanib was 200 mg/mq b.i.d. in combination with full doses of paclitaxel and carboplatin, and dose-limiting toxicities were liver enzyme elevations, thrombocytopenia, abdominal pain, and rash. Partial responses were observed in 26.9% of patients, and stable disease was observed in 38.5% of patients.

These trials confirm that splitting the total daily dose into two daily administrations increases the total daily exposure without additional toxicity. They also showed that 200 mg b.i.d. of nintedanib is the recommended dose for continuous daily treatment in combination with standard-dose pemetrexed or carboplatin and paclitaxel for patients with advanced or metastatic NSCLC (11, 12).

In all these phase I studies, nintedanib revealed a similar adverse event profile with respect to fatigue and gastrointestinal adverse events as compared with other VEGFR TKIs. The predominant adverse events were nausea, diarrhea, vomiting, abdominal pain, and fatigue of low to moderate intensity during the first 2 months of therapy. Dose-limiting toxicities were dose-dependent hepatic enzyme elevations that were reversible after discontinuation of nintedanib treatment. Only in few patients, liver enzyme elevations were accompanied by a simultaneous increase in bilirubin. In general, common terminology criteria for adverse events (version 3.0) grade 3 liver enzyme increases were reported in the dose groups of 250 mg twice daily or higher. Severe grade 4 liver enzyme elevations were observed only occasionally, and they were fully reversible within 2 weeks to treatment discontinuation or dose reduction. Fatigue was also reported of a mild-to-moderate intensity, instead in the trial of nintedanib with pemetrexed it was reported as the most relevant dose-limiting toxicity. There were no drug-related bleeding events. Hypertension or thromboembolic events were rare and did not suggest an increased frequency as a consequence of therapy with nintedanib. There was no increase in hematologic toxicity observed when nintedanib was combined with chemotherapy. Unlike some other oral angiogenesis inhibitors, nintedanib did not seem to cause relevant skin abnormalities and no hand-foot syndrome was observed.

## Phase II Studies

Reck et al. conducted a phase II double-blinded, two-arm, randomized monotherapy trial with nintedanib (13). Patients with locally advanced or metastatic relapsed NSCLC of any histology after failure of first- or second-line chemotherapy with an Eastern Cooperative Oncology Group (ECOG) performance status (PS) 0–2 were randomized to continuous 150 or 250 mg b.i.d. nintedanib treatment until disease progression. Progression-free survival (PFS) and overall response rate were primary end points. Secondary end points included pharmacokinetic profiles of nintedanib, safety, and overall survival (OS). There was no significant difference in the PFS and the OS between the two groups. The results of this trial demonstrate that nintedanib in patients with ECOG 0–1 reaches effectiveness comparable to historical phase II data of other VEGFR inhibitors in a similar patient population: median PFS was 2.9 months with nintedanib, 2.8 months with sunitinib (21), 2.8 months with sorafenib (22), 2.6 months with vandetanib (23), and 3.5 months with vatalanib (24). The toxicity profile in this study was similar to that seen in phase I trials (17, 18). The majority of the adverse events were mild-to-moderate gastrointestinal symptoms with reversible hepatic toxicity. Tolerability was comparable between the two doses, with the exception of a higher frequency of liver enzyme elevations in the higher dose group.

## Phase III Studies

Two randomized prospective clinical trials have been conducted to evaluate the efficacy of nintedanib in patients with advanced NSCLC. The LUME-Lung 1 was a large multicenter double-blind, placebo-controlled, phase III trial randomizing patients with NSCLC to second-line docetaxel plus placebo (*n* = 659) or docetaxel plus nintedanib (*n* = 655) (14). The primary end point was PFS by central independent review, and the secondary end point was OS; additional secondary end points included investigator-assessed PFS, tumor response by central review and investigator assessment, safety, and patient-reported quality of life (QoL). Patients were randomized in a 1:1 ratio to investigational arm of nintedanib 200 mg b.i.d. plus standard docetaxel therapy 75 mg/m^2^ vs. placebo plus standard docetaxel therapy. A total of 1,314 patients were randomized: 655 assigned to experimental arm and 659 to standard arm. Patients were stratified by histology, ECOG PS, prior bevacizumab treatment, and the presence of brain metastases allowed if stable. Exclusion criteria were as follows: previous treatment with docetaxel or other VEGF inhibitors therapy (with the exception of bevacizumab), active and unstable brain metastasis or radiographic evidence of cavitary or necrotic tumors. Baseline demographics were well balanced between both arms. In this trial, the addition of nintedanib to docetaxel significantly improved PFS in the total study population (median 3.4 months [95% CI: 2.9–3.9] vs. 2.7 months [2.6–2.8]; hazard ratio (HR): 0.79 [95% CI: 0.68–0.92], *p* = 0.0019). The benefit in PFS was consistent, regardless of gender, age, ethnicity, or PS.

Moreover, the addition of nintedanib improved median OS in patients with adenocarcinoma (12.6 months [95% CI: 10.6–15.1] vs. 10.3 months [95% CI: 8.6–12.2]; HR: 0.83 [95% CI: 0.70–0.99], *p* = 0.0359). The prolongation of OS was consistent with the improvement of 1-year survival rate from 45 up to 53% and 2-year survival rate from 19 up to 26%. OS was also increased in patients with adenocarcinoma histology who progressed within 9 months after start of first-line treatment (median OS increased from 7.9 to 10.9 months corresponding to a HR of 0.75 and *p* value of 0.0073) and in patients refractory to first-line chemotherapy. In this group of poor prognosis patients, an advantage of more than 3 months was observed with the addition of nintedanib to docetaxel compared to docetaxel alone (9.8 vs. 6.3 months, HR of 0.62, *p* = 0.0246). There was no difference in OS in the total study population (median 10.1 months [95% CI: 8.8–11.2] vs. 9.1 months [8.4–10.4]; HR: 0.94 [95% CI: 0.83–1.05], *p* = 0.2720) and in patients with squamous cell carcinoma between both arms. Finally, the investigation of the interaction between treatment and tumor burden showed that a greater tumor burden was associated with a greater treatment effect for docetaxel and nintedanib. In addition, a significant improvement in disease control rate (60.2 vs. 44%) in favor of nintedanib plus docetaxel was observed in adenocarcinoma patients. Adverse events more common in the docetaxel plus nintedanib group than the docetaxel plus placebo group were as follows: diarrhea (all grades: 42.3 vs. 21.8%; grade ≥ 3 6.6 vs. 2.6%), increases in alanine aminotransferase (all grades, 28.5 vs. 8.4%; grade ≥ 3 7.8 vs. 0.9%), nausea (all grades, 24.2 vs. 18.0%; grade ≥ 3, 0.8 vs. 0.9%), increases in aspartate aminotransferase (all grades, 22.5 vs. 6.6%; grade ≥ 3, 3.4 vs. 0.5%), decreased appetite (all grades, 22.2 vs. 15.6%; grade ≥ 3, 1.4 vs. 1.2%), and vomiting (all grades 16.9 vs. 9.3%; grade ≥ 3 0.8 vs. 0.5%). There was no statistically significant increase in the incidence of bleeding and hypertension events by the addition of nintedanib (25). Moreover, the significant OS benefit observed with the addition of nintedanib to docetaxel therapy was achieved with no detrimental effect on patient self-reported QoL, with significant reductions in some pain items with nintedanib vs. placebo (26).

LUME-Lung 2 was a multicenter, randomized, double-blinded phase III study that investigated the efficacy and safety of nintedanib in combination with pemetrexed vs. placebo plus pemetrexed in patients with locally advanced or metastatic non-squamous NSCLC with relapse or failure after chemotherapy (15). A total of 713 patients were randomized 1:1 to experimental arm (353 patients) and to standard arm (360 patients). The primary end point was centrally reviewed PFS, the secondary end points were OS, investigator-assessed PFS, objective response rate (ORR), safety, and QoL. The study enrolled patients with ECOG PS 0–1 without active brain metastases, cavitary or necrotic tumors, and clinically significant hemoptysis, not previously treated with VEGF inhibitors (except bevacizumab). Baseline patient characteristics were balanced between both arms for age, gender, PS, histology type, and prior bevacizumab treatment. All randomized patients were included in the intention-to-treat (ITT) population. The study was designed to have 90% power to demonstrate a significant (27.5%) improvement in PFS with a HR of 0.78 after 713 PFS events.

The analysis suggested that the primary end point of centrally assessed PFS would likely not be met; however, there were no safety concerns. Ongoing patients were unblinded and follow-up was continued per protocol. Analysis of the primary end point PFS by independent central review was conducted after 498 events had occurred, and analysis of the secondary end point OS was conducted after 436 events had occurred. The primary end point of this phase III trial was met even though the study was stopped prematurely. ITT analysis of the primary end point showed that treatment with nintedanib plus pemetrexed resulted in a significant prolongation of PFS compared with placebo plus pemetrexed (4.4 vs. 3.6 months with a HR of 0.83 and a *p* value of 0.04). Disease control rate was also increased significantly in nintedanib-treated group (61 vs. 53%, with an odds ratio of 1.37 and a *p* value of 0.039). No difference in OS was seen between the arms. There was no increase in serious side effects in the combination arm. However, there was an increase in the incidence of diarrhea and elevated liver enzymes, each of which were reversible. There was no difference between the arms in terms of the incidence of hypertension, bleeding, thrombosis, mucositis, or neuropathy.

## Nintedanib in Other Tumors

Due to the important rule of angiogenesis pathways identified in cancer development, Nintedanib has also been evaluated in other tumors (Table 2).

**Table 2:** **Studies with nintedanib in other tumors**.

Phase and reference	Line of treatment	Setting	#Patients	Treatment	Results

Systemic treatment					
II; Han et al. (27)	≥2nd	Relapsed small cell lung cancer	24	Nintedanib 200 mg × 2/day	Objective response rate = 5%
					Hepatic enzyme elevation 86%
II; Palmer et al. (28)	1st	Unresectable HCC	93	Nintedanib 200 mg × 2/day vs. sorafenib	Time to progression: 5.5 vs. 3.8 months
					Overall survival (OS): 11.9 vs. 11.4 months
					Comparable toxicities
II; Eisen et al. (29)	1st	Advanced RCC	96	Nintedanib 200 mg × 2/day vs. sunitinib	Progression-free survival (PFS) at 9 months 43.1 vs. 45.2%
					OS: 20.4 vs. 21.2 months
					Comparable toxicities
II; Norden et al. (30)	≥2nd	Recurrent glioblastoma	36	Nintedanib 200 mg × 2/day	No responses
					PFS at 3 (prior bevacizumab) and 6 (no prior bevacizumab) months = 0%
II; Droz et al. (31)	≥2nd	Prostate cancer	81	Nintedanib 150 or 250 mg × 2/day	PSA decrease under 50% = 5.6%
					PFS: 73.5–76 days
II; Van Cutsem et al. (32)	1st	Colorectal cancer	126	mFOLFOX6 + nintedanib 200 mg × 2/day or bevacizumab 5 mg/kg every 14 days	PFS at 9 months: 62.1 vs. 70.2%
II; Ledermann et al. (33)	≥2nd	Ovarian cancer	83	Nintedanib 250 mg × 2/day vs. placebo for up to 9 months as maintenance following chemotherapy	% of patients progression free at 36 weeks: 16.3 vs. 5%
					Grade 3 or 4 hepatotoxicity 51.2 vs. 7.5%
III, AGO-OVAR 12; du Bois et al. (34)	1st	Ovarian cancer	1,366	Carboplatin (AUC 5/6) + paclitaxel (175 mg/mq) d1 + nintedanib 200 mg × 2/day or placebo days 2–21 q21 × 6 cycles → nintedanib or placebo maintenance for up to 2 years	PFS: 17.2 vs. 16.6 months, hazard ratio: 0.84, *p* = 0.024
					G3 diarrhea 21 vs. 2%, G4 neutropenia 22 vs. 16%, G4 thrombocytopenia 6 vs. 2%

*PSA, prostate-specific antigen*.

### Small Cell Lung Cancer

A phase II study evaluated nintedanib activity in 24 patients with small cell lung cancer (SCLC) relapsed after one or two lines of chemotherapy or chemoradiotherapy (27). Eight patients received only one prior chemotherapy. Nintedanib was administered at 200 mg twice daily until disease progression or toxicity. ORR, the primary end point, was 5% [95% CI: 0.1–22.8]. Median PFS was 1 month and OS was 9.8 months. The most frequent drug-related adverse events included hepatic enzyme elevation (86%), anemia (73%), anorexia (59%), and nausea (50%). Most toxicities were mild and manageable. Grade 3 hepatic enzyme elevation occurred in five patients (23%). The authors concluded that nintedanib exhibited only a modest activity in relapsed or refractory SCLC.

### Hepatocellular Carcinoma

A phase II study was designed to compare safety and activity of nintedanib 200 mg b.i.d. vs. sorafenib 400 mg b.i.d. in 93 patients with unresectable hepatocellular carcinoma and Child-Pugh A score, randomized in a 2:1 ratio (28). Time to progression, the primary objective, was comparable between nintedanib and sorafenib (median 5.5 vs. 3.8 months; HR: 1.05 [95% CI: 0.63–1.76]). Median OS was 11.9 vs. 11.4 months, respectively (HR: 0.88 [95% CI: 0.52–1.47]). More patients treated with sorafenib had grade ≥ 3 adverse events (68 vs. 90%). Toxicities leading to dose reduction were higher with sorafenib (19 vs. 42%), whereas side effects leading to drug discontinuation were higher with nintedanib (45 vs. 23%). Rash was reported in >15% of patients only in the sorafenib arm.

### Renal Cell Carcinoma

A phase II study evaluated activity and tolerability of first-line nintedanib 200 mg twice daily vs. standard sunitinib in 96 patients with advanced renal cell carcinoma, randomized in a 2:1 ratio (29). The trial would also test possible electrocardiographic changes, particularly in QTc, during nintedanib assumption. PFS at 9 months, the primary objective, was 43.1 vs. 45.2% (*p* = 0.85) for nintedanib vs. sunitinib. Median OS was 20.4 vs. 21.2 months (HR: 0.92; 95% CI: 0.54–1.56; *p* = 0.76). Toxicities were comparable between the two treatments. Nintedanib was associated with lower incidences of some adverse events typical of antiangiogenic TKIs, such as hypertension, hypothyroidism, hand-foot syndrome, cardiac disorders, and hematological abnormalities.

### Glioblastoma

Activity of nintedanib was also explored in patients with recurrent glioblastomas, but the results were disappointing. In a phase II study, 36 patients, stratified based on prior bevacizumab, received nintedanib 200 mg twice daily (30). There were no responses, and PFS at 3 (prior bevacizumab) and 6 (no prior bevacizumab) months was 0%.

### Castration-Resistant Prostate Cancer

Modest activity was noted with nintedanib 150 or 250 mg twice daily in 81 castration-resistant prostate cancer patients pretreated with docetaxel chemotherapy (31). Only 5.6% of patients treated with nintedanib 250 mg obtained a prostate-specific antigen (PSA) decrease of at least 50%. Median PFS was 73.5 and 76 days with nindetanib 150 and 250 mg, respectively. Toxicities included gastrointestinal disorders, asthenia, hypertension, and reversible elevated transaminases. A phase I trial tested nintedanib in association with docetaxel (75 mg/m^2^ every 3 weeks) and prednisone in castration-resistant prostate cancer patients (19), suggesting the dose of 200 mg twice daily for future investigations. Among 19 assessable patients, 13 (68.4%) showed a ≥50% reduction in PSA levels from baseline. Pharmacokinetic analysis showed no interactions between nintedanib and docetaxel/prednisone.

### Metastatic Colorectal Cancer

A phase I/II study tested nintedanib + mFOLFOX6 or bevacizumab + mFOLFOX6 in the first-line treatment of patients with advanced colorectal cancer (32). In the phase II of the study, nintedanib was given at 200 mg twice daily. Overall, 126 patients were randomized in a 2:1 ratio into the nintedanib vs. bevacizumab arm. PFS at 9 months, the primary objective, was 62.1 vs. 70.2%, while objective response was 63.5 vs. 56.1%. The incidence of serious adverse events was 37.6% with nintedanib and 53.7% with bevacizumab. The pharmacokinetics of nintedanib and the components of mFOLFOX6 were unaffected by their combination.

### Ovarian Cancer

A randomized phase II study was conducted with nintedanib in 83 relapsed ovarian cancer patients. In this study, women treated with nintedanib as maintenance therapy at 250 mg twice daily for up to 9 months after chemotherapy were less likely to experience disease progression compared to those treated with placebo (33). At 36 weeks, 16.3% of women taking nintedanib were progression free, compared to 5% of those taking placebo (HR: 0.65; 95% CI: 0.42–1.02; *p* = 0.06). Two patients continued nintedanib for another year or more. More patients on nintedanib experienced diarrhea, nausea, or vomiting (no grade 4). There was a higher rate of grade 3 or 4 hepatotoxicity in patients on nintedanib (51.2%) compared with patients on placebo (7.5%; *p* < 0.001).

LUME-Ovar 1, also named AGO-OVAR 12, is a phase III study testing association of first-line chemotherapy plus nintedanib or placebo in patients with advanced ovarian carcinoma (34). The trial recruited 1,366 patients with FIGO IIB-IV ovarian carcinoma and primary debulking surgery to receive in a 2:1 ratio of six cycles of carboplatin (AUC 5 or 6 mg/dl/min) and paclitaxel (175 mg/m^2^) on day 1 every 3 weeks plus nintedanib 200 mg twice daily on days 2–21 of each cycle or placebo. The biological agent or placebo were given for up to 120 weeks. Primary end point was PFS by investigator assessment in the ITT population. As a result, 53% of 911 patients in the nintedanib group experienced disease progression or death compared with 58% of 455 patients in the placebo group. Median PFS was significantly longer with nintedanib than placebo (17.2 months [95% CI: 16.6–19.9] vs. 16.6 months [13.9–19.1]; HR: 0.84 [95% CI: 0.72–0.98]; *p* = 0.024). The most common adverse events were gastrointestinal, such as grade 3 diarrhea in 21% of patients receiving nintedanib vs. 2% in the placebo group, and hematological (neutropenia of grade 3 in 20% and grade 4 in 22% of patients receiving nintedanib vs. 20 and 16% in the placebo group, respectively; thrombocytopenia 12 and 6% vs. 5 and 2%; anemia 12 and 1% vs. 6 and 1%). Serious adverse events were reported in 42% with nintedanib and 34% with placebo; 3% of patients receiving nintedanib experienced serious adverse events associated with death compared with 4% in the placebo group.

## Ongoing Clinical Studies with Nintedanib

Nintedanib is currently under investigation in various types of tumor (Table 3). In neoadjuvant setting, a phase I study is evaluating the safety of nintedanib in combination with cisplatin and docetaxel before surgery in patients with stages I–III NSCLC [http://ClinicalTrials.gov: NCT02225405]. LUME-Meso is a randomized double-blind phase II/III study testing safety and efficacy of nintedanib in 537 naïve patients with unresectable pleural mesothelioma (35). Treatment consists of six courses of chemotherapy with cisplatin 75 mg/m^2^ and pemetrexed 500 mg/m^2^ on day 1 plus nintedanib 200 mg b.i.d. on days 2–21 of each cycle or placebo. Following maintenance with biologic agent, placebo is given to patients with controlled disease. Primary end point is PSF, and secondary end points include OS, objective response, and disease control rate. Preliminary results are expected in 2019. LUME-Colon 1 is a double-blind randomized phase III study evaluating monotherapy with nintedanib and best supportive care (BSC) vs. placebo and BSC in patients with refractory advanced colorectal cancer pretreated with standard chemotherapies and biologic agents (36). ECOG PS 0–1 and life expectancy of minimum 12 weeks are required. Estimated accrual is of 764 patients. Prior regorafenib is allowed. Patients are stratified based on previous regorafenib, time from onset of metastatic disease to randomization (less or more than 24 months), and region. Nintedanib is administered at 200 mg twice daily vs. placebo in a 1:1 randomization. Primary outcomes are PFS by central review assessment and OS, with objective tumor response and disease control as secondary end points. Other assessments include frequency and severity of adverse events, changes in laboratory parameters, health-related QoL, and biomarker analyses to better define predictiveness of response and drug resistance mechanisms. Final results are soon expected. LUME-Colon 2 is a phase II study assessing nintedanib alone or in combination with capecitabine in patients with refractory metastatic colorectal cancer after failure of at least two lines of standard treatment. Primary end point is PFS. Estimated enrollment is 100 patients. Results are expected in 2017 [http://ClinicalTrials.gov: NCT02780700]. A phase II study is testing first- or second-line docetaxel ± nintedanib in patients with HER2-negative metastatic or locally recurrent breast cancer. Docetaxel 75 mg/m^2^ every 3 weeks could be increased to 100 mg/m^2^ in the arm without nintedanib. Nintedanib is administered at 200 mg twice daily from day 2 of each cycle. Primary objective is PFS. Secondary end points are response rate, OS, QoL, and pharmacokinetic analyses. Estimated enrollment is 252 patients, and results are soon expected [http://ClinicalTrials.gov: NCT01658462].

**Table 3:** **Ongoing studies with nintedanib**.

Phase	Line of treatment	Setting	#Patients	Treatment	Endpoints

Systemic treatment					
I	Neoadjuvant	Resectable non-small cell lung cancer stage IB–IIIA	45	Cisplatin + docetaxel + nintedanib	Major pathologic response rate
					Toxicity of nintedanib given with cisplatin and docetaxel
II/III	1st	Unresectable pleural mesothelioma	537	Cisplatin-pemetrexed + nintedanib or placebo → nintedanib or placebo maintenance	Progression-free survival (PFS)
III	Advanced	Advanced colorectal cancer	764	Monotherapy with nintedanib 200 mg × 2/day vs. placebo (prior regorafenib allowed)	PFS and overall survival
III	Advanced	Advanced colorectal cancer	100	Nintedanib alone or in combination with capecitabine	PFS
II	1st or 2nd	Advanced HER2-negative breast cancer	252	Docetaxel d1 ± nintedanib 200 mg × 2/day, days 2–21	PFS
I	Advanced	Refractory solid tumors	18	Nintedanib + pembrolizumab	Maximum tolerated dose of nintedanib

Finally, an ongoing phase I trial is testing nintedanib and pembrolizumab in refractory solid tumors patients to define the toxicity profile of such combination [http://ClinicalTrials.gov: NCT02856425].

## Discussion and Conclusion

Two randomized phase III clinical trials have evaluated to date the efficacy of nintedanib in patients with advanced NSCLC. The LUME-Lung 1 trial have showed, for the first time, an OS benefit in patients with advanced NSCLC from the addition of a targeted agent to chemotherapy in the second-line setting. In this trial, the addition of nintedanib to docetaxel significantly improved median OS in patients with adenocarcinoma histology (from 10.3 to 12.6 months), with a greater advantage in patients who progressed within 9 months after start of first-line treatment (from 7.9 to 10.9 months) and in patients who were most refractory to first-line chemotherapy (from 6.3 to 9.8 months). Moreover, nintedanib plus docetaxel improved PFS and disease control in the total study population. These results were partially confirmed by the LUME-Lung 2 trial that, despite early closure, showed that nintedanib plus pemetrexed resulted in a significant prolongation of PFS and disease control rate, while no difference in OS was seen between the arms, probably due to the final low power of the study. On these bases, the combination of docetaxel and nintedanib can be considered a new option for the second-line treatment for patients with advanced NSCLC with adenocarcinoma histology (37).

However, there are several issues that need to be addressed, including the following: (a) how to improve the tolerability profile of the combination of docetaxel and nintedanib; (b) the role of nintedanib in other settings, such as first line and neoadjuvant; (c) the feasibility of combining nintedanib with other drugs; (d) the activity of nintedanib in other tumors; and (e) the identification of predictive factors.

The most frequent adverse events of nintedanib as single agent were nausea, diarrhea, vomiting, increases in liver enzymes, and fatigue, generally of low to moderate intensity, while hypertension or thromboembolic events were rare. Combination of nintedanib with docetaxel revealed a similar toxicity profile as compared to nintedanib monotherapy, except for docetaxel-related toxicities. Chemotherapy with docetaxel 75 mg/mq administered once every 3 weeks has been proven to be a reasonable therapeutic choice for the second-line treatment of patients with advanced NSCLC, but myelosuppression is extremely frequent and severe: weekly scheduling of docetaxel has demonstrated to improve the toxicity profile of the drug in pretreated NSCLC patients without decreasing antitumor activity (38). Therefore, the addition of nintedanib to weekly docetaxel could be an attractive schedule to maintain the therapeutic efficacy of the combination with a better toxicity profile. An Italian multicenter, prospective, open-label study with two “cohorts” is evaluating the efficacy and safety profile of nintedanib plus docetaxel in patients with non-squamous NSCLC in stage IIIB/IV with two different combination schedules, including a weekly schedule of docetaxel (SENECA trial): the results of this trial should answer the question of the feasibility and activity of the combination of nintedanib with weekly docetaxel.

In other settings, a phase I study investigated nintedanib combined with paclitaxel (200 mg/mq) and carboplatin (AUC 6 mg/ml/min), in first-line setting in 26 patients with advanced NSCLC (21). This combination was well tolerated, without drug-to-drug interactions and demonstrated promising preliminary efficacy in patients with advanced NSCLC, supporting further investigation in patients with NSCLC. In neoadjuvant setting, a phase I study is evaluating the safety of nintedanib in combination with cisplatin and docetaxel before surgery in patients with stage I–III NSCLC.

A number of studies are evaluating the feasibility of the combination of nintedanib and other classes of drugs, including angiogenesis inhibitors such as bevacizumab, EGFR inhibitors such as afatinib, and immune checkpoint inhibitors such as pembrolizumab. The good safety profile of the drug allows to use nintedanib also in special populations, such as elderly patients, in combination with other chemotherapeutic agents: the VENUS-1 and VENUS-2 are dose-escalation trials to evaluate the feasibility of the combination of nintedanib with vinorelbine or with carboplatin and vinorelbine in elderly patients with advanced NSCLC.

Ongoing studies will clarify the activity of nintedanib in other tumors, including mesothelioma, colon, breast, ovarian, cervix, pancreatic cancer, and HCC.

Identifying molecular biomarkers that can predict a response to nintedanib remains an important goal to maximize the clinical benefit of this agent. A phase II study is ongoing to examine the value of FGFR1 gene amplification as a predictor of nintedanib efficacy in patients with squamous cell NSCLC [http://ClinicalTrials.gov: NCT01948141]. Additional studies are planned that include translational approaches to identify more detailed mechanisms of action for nintedanib.

In conclusion, nintedanib is an effective second-line treatment in combination with docetaxel for patients with lung adenocarcinoma, also refractory to first-line chemotherapy. Future challenges are to indentify predictive factors to help the decision of using antiangiogenic agents in patients.

## Author Contributions

All the authors have made substantial contributions to the conception, drafting, and revision of the article and approved the final version.

## Conflict of Interest Statement

The authors declare that the research was conducted in the absence of any commercial or financial relationships that could be construed as a potential conflict of interest.
